# A Triplet Network Fusing Optical and SAR Images for Colored Steel Building Extraction

**DOI:** 10.3390/s24010089

**Published:** 2023-12-23

**Authors:** Xiaoyong Zhang, Shuo Yang, Xuan Yang, Cong Li, Yue Xu

**Affiliations:** 1 Beijing Key Laboratory of High Dynamic Navigation, Beijing Information Science and Technology University, Beijing 100101, China; zhangxy@bistu.edu.cn (X.Z.); 2021020378@bistu.edu.cn (S.Y.); 2 State Key Laboratory of Remote Sensing Science, Aerospace Information Research Institute, Chinese Academy of Sciences, Beijing 100094, China; licong@aircas.ac.cn; 3 China Remote Sensing Satellite Ground Station, Aerospace Information Research Institute, Chinese Academy of Sciences, Beijing 100094, China; yangxuan@radi.ac.cn

**Keywords:** colored steel building extraction, data fusion network, semantic segmentation, urban monitoring, SAR imagery enhancement, Beijing–Tianjin–Hebei metropolitan region

## Abstract

The identification of colored steel buildings in images is crucial for managing the construction sector, environmental protection, and sustainable urban development. Current deep learning methods for optical remote sensing images often encounter challenges such as confusion between the roof color or shape of regular buildings and colored steel structures. Additionally, common semantic segmentation networks exhibit poor generalization and inadequate boundary regularization when extracting colored steel buildings. To overcome these limitations, we utilized the metal detection and differentiation capabilities inherent in synthetic aperture radar (SAR) data to develop a network that integrates optical and SAR data. This network, employing a triple-input structure, effectively captures the unique features of colored steel buildings. We designed a multimodal hybrid attention module in the network that discerns the varying importance of each data source depending on the context. Additionally, a boundary refinement (BR) module was introduced to extract the boundaries of the colored steel buildings in a more regular manner, and a deep supervision strategy was implemented to improve the performance of the network in the colored steel building extraction task. A BR module and deep supervision strategy were also implemented to sharpen the extraction of building boundaries, thereby enhancing the network’s accuracy and adaptability. The results indicate that, compared to mainstream semantic segmentation, this method effectively enhances the precision of colored steel building detection, achieving an accuracy rate of 83.19%. This improvement marks a significant advancement in monitoring illegal constructions and supporting the sustainable development of the Beijing–Tianjin–Hebei metropolitan region.

## 1. Introduction

In urban development, colored steel buildings are prominent due to their construction from color-coated steel plates, offering lightweight, robust, and durable properties. These buildings are extensively utilized for temporary structures, playing a significant role in urban development [1,2]. However, unauthorized colored steel constructions can disrupt city planning, pose safety risks, and have negative environmental impacts [3,4]. Accurate identification and monitoring of these buildings are essential, facilitating the enforcement of urban regulations and the sustainable management of urban growth. By effectively tracking and regulating the spread of colored steel buildings, urban authorities can ensure compliance with planning laws and maintain ecological balance.

The advent of high-resolution remote sensing imagery, bolstered by advances in computer technology, has enabled the capture of intricate details in urban structures, including geometric and textural features [5,6]. Historically, colored steel buildings have been identified and analyzed using optical data. For instance, Ma et al. [7,8] manually interpreted remote sensing images from QuickBird-2 (2005) and Gaofen-2 (2017) to map colored steel buildings in Lanzhou City’s Anning District, examining their spatial–temporal patterns and clustering characteristics. This manual approach, however, is labor-intensive for large-scale applications. Alim et al. [9] utilized Sentinel-2A/B MSIL2A data with a 10 m resolution and introduced six spectral indices to differentiate materials of blue- and red-colored steel buildings. Similarly, Li et al. [10] employed GF-1 remote sensing imagery, selecting optimal scales for image segmentation and formulating rules for knowledge extraction based on color, spectral, and geometric features, leading to an automated decision-tree-based information extraction process. Despite these efforts, traditional methods face challenges, such as setting appropriate threshold values, the presence of intra-class mixed pixels, and extensive shadows in remote sensing images, which degrade the accuracy of extraction results and necessitate significant manual correction. Consequently, pixel-based classification techniques in remote sensing fall short in addressing the demands for large-scale extraction of colored steel buildings.

The integration of deep learning into remote sensing for image segmentation has seen considerable progress in recent years, maturing the application in this domain [11,12,13,14]. Deep learning methods surpass traditional techniques with their potent feature extraction, minimal human supervision, and robust algorithmic performance [15,16,17]. These advances have led to the adoption of image segmentation networks for the identification of steel-structured buildings. For example, Shen Shunfa and colleagues adapted Segnet and U-net models, introducing improvements to U-net for better low-level feature retention, thus refining the accuracy of colored steel building extraction [18]. Li [19] enhanced the detection of colored steel buildings through an advanced MaskR-CNN instance segmentation algorithm, integrating a CBAM convolutional attention mechanism to better focus on target regions. Despite these developments, certain issues remain unresolved, such as limited model generalization, irregular boundaries in the segmentation output, and the challenge of distinguishing colored steel buildings from conventional structures due to similar roof characteristics in optical images.

The widespread application of multi-source data fusion methods has become common practice [20,21]. These fusion methods aim to capitalize on the complementary strengths of different sensor modalities, enhancing the overall information content and improving the robustness of remote sensing applications. Notably, optical and SAR image fusion methods have been extensively explored due to their distinct advantages [22,23]. Synthetic aperture radar (SAR) data have the capability to detect and differentiate metallic materials, and are less susceptible to atmospheric conditions such as cloud cover compared to optical data [24,25,26]. Colored steel buildings and residential structures have roofs made of metal and concrete, respectively. Due to the distinct electromagnetic properties of metal and non-metal objects, SAR imagery typically exhibits different scattering patterns. Metal objects tend to generate prominent echoes due to their higher electrical conductivity, while non-metal objects may exhibit more scattering and absorption. Therefore, the introduction of SAR data is considered to address the limitations of optical data in reflecting the material composition of colored steel buildings. This approach enables the accurate identification of colored steel buildings over large areas. Li et al. employed optical and SAR images to design a multimodal cross-attention network (MCANet) for land use classification. The inputs to the network are independent optical and SAR images. Li’s team proposed an innovative multimodal cross-attention module (MCAM) that adeptly captures the positional relationships within individual data source feature maps. This module effectively interacts within a two-dimensional space, incorporating both SAR and optical image feature maps [27].

In order to better fuse optical and SAR images, we first designed a three-branch multi-source data fusion network for extracting colored steel buildings using 0.8 m high-resolution remote sensing images and SAR image fusion. By splicing the optical and SAR channels into a third branch, the network can utilize the information from both data sources more comprehensively and reduce the interference between them. We also devised an attention module tailored for our network’s three-branch input, dynamically adjusting attention weights on each channel. This allows the network to adaptively emphasize or suppress information from different channels to better suit various colored steel building extraction scenarios. Among various methods, conditional random field (CRF) is often employed due to its robust mathematical formulation. Deeplab [28] utilizes dense CRF [29], a CRF variant on a fully connected graph, as a post-processing method after CNN. However, with Deeplabv3 fusing image-level features into the ASPP module [30], the use of CRF may increase and the computational overhead will be large, so we introduced a BR module similar to the residual structure to alleviate the problem of gradient vanishing, which helps to optimize the boundaries to a certain extent. In summary, this method of integrating optical and SAR data is expected to make up for the lack of optical data in colored steel building extraction, providing new ideas and possibilities to further improve the accuracy of building material identification and building extraction.

In summary, the contributions of this study are as follows:

(1) We propose a network structure for multi-source data fusion to better fuse GF-2 and SAR features, which performs well for large-scale colored steel building extraction compared to optical data;
(2) A multimodal three-branch attention module is proposed on the three-branch network for capturing SAR and GF-2 features in both spatial and channel dimensions, allowing the network to learn how important each branch is in different situations;
(3) Experiments show that by introducing a deep supervision strategy, the network helps to learn feature representations more efficiently, which improves the generalization ability of the model.

## 2. Materials and Methods

### 2.1. Study Area

**Figure 1:**
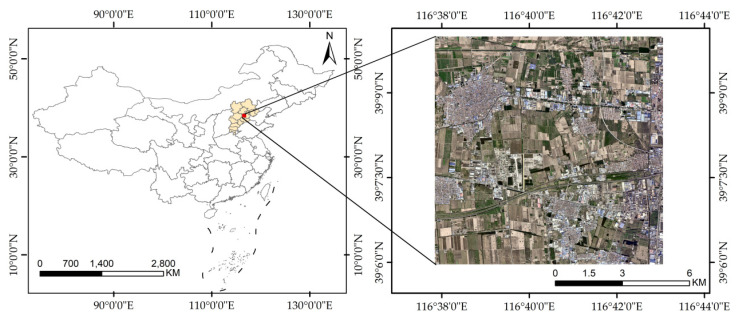
Location of the research area and local imagery. The left image depicts the specific location of the research area; the right image shows a GF-2 image of a particular region in Langfang City within the research area, which includes numerous colored steel structures.

The Beijing–Tianjin–Hebei region, highlighted in Figure 1, was selected for this study on colored steel building extraction due to its significance in China’s urbanization process. Spanning approximately 225,000 km^2^, this urban agglomeration includes Beijing, Tianjin, Hebei Province, and Anyang City in Henan Province. This region, being the political, cultural, and economic hub of China, sees extensive use of colored steel buildings owing to their rapid assembly and adaptability, catering to the swift urban growth and interim construction demands. With a dense population and pressing requirements for housing and infrastructure, the Beijing–Tianjin–Hebei area underscores the necessity for regularized and lawful urban development, making it an apt focus for the study of colored steel construction practices.

### 2.2. Data Sources

We utilized data from two satellite sources: China’s Gaofen-2 (GF-2) and the European Space Agency’s Sentinel-1.

The GF-2 satellite represents China’s foray into high-definition optical remote sensing technology, with a spatial resolution of less than 1 m, and was launched via the Long March 4B rocket on 19 August 2014. It carries a multispectral camera with one 4 m resolution and two 1 m resolution panchromatic cameras, covering an observational swath of 60 km. This satellite is integral to projects on land surveillance and urban development planning [31,32].

The Sentinel-1 satellite, part of the European Space Agency’s Copernicus Global Monitoring for Environment and Security (GMES) program, is equipped with C-band SAR instruments capable of obtaining imagery under almost any weather condition, during day or night [33]. SAR technology is particularly adept at cloud penetration, ensuring continuous surface data capture. For this research, the Sentinel-1 satellite’s dual-polarization ground range detected (GRD) data products were chosen for their 10 m spatial resolution. The vertical–vertical (VV) polarization mode is utilized for its enhancement of geometric shapes, and the vertical–horizontal (VH) polarization is used for its detailed surface feature representation. These capabilities make Sentinel-1 invaluable for a variety of applications, including land alteration studies and disaster monitoring.

### 2.3. Data Preparation and Sample Construction

In this study, the focus was on colored steel buildings, which are predominantly made from metal and are coated with colored steel, with blue and red being the most common roof colors, along with occasionally, gray and white [34]. These structures vary in size and are often rectangular or composite rectangular shapes; the roofs are generally smooth but they can be confused with residential buildings in remote sensing imagery, leading to classification errors. To mitigate this issue, our research incorporates SAR imagery to complement the dataset, enhancing the model’s ability to distinguish colored steel buildings from other structures and improving the accuracy of the semantic segmentation process.

**Figure 2:**
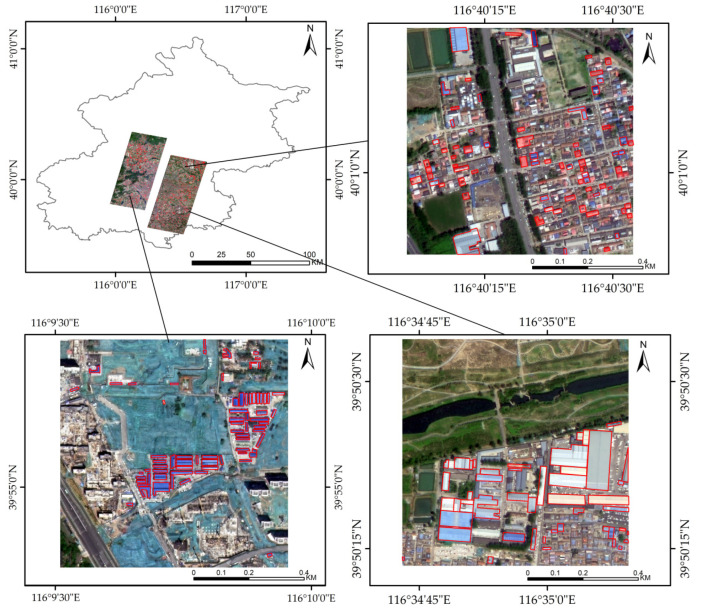
Sample production scope and local images.

We utilized a combination of high-resolution and SAR imagery to isolate colored steel buildings, which are discernible by their shape, size, color, and other features. The process began with acquiring remote sensing data from the GF-2 and Sentinel-1 satellites for the designated study area. The initial steps involved converting bit depth, correcting geometric distortions, and detecting and relegating cloudy images for mosaic construction. In 2019, detailed manual annotation of 81,419 sample plots within Beijing’s densely populated colored steel building zones was conducted, as shown in Figure 2. Labeling was finalized through vector-to-raster conversion in ArcGIS 10.8 software. For experimental purposes, the blue–green–red band from GF-2 and the 10 m resolution Sentinel-1 images were standardized to a 0.8 m resolution. Due to the extensive size of the images, they were sectioned into 1024 × 1024 pixel frames before being input into the neural network. The samples were then categorized into training, validation, and test groups at an 8:1:1 ratio, yielding 9539 training, 1586 validation, and 1236 test samples. To boost the model’s ability to generalize, data augmentation tactics were employed on the training set, including image flipping and random variations in brightness and color, as well as cropping, to create a robust dataset for neural network training.

### 2.4. Methods

#### 2.4.1. Network Structure for Multi-Source Data Fusion

**Figure 3:**
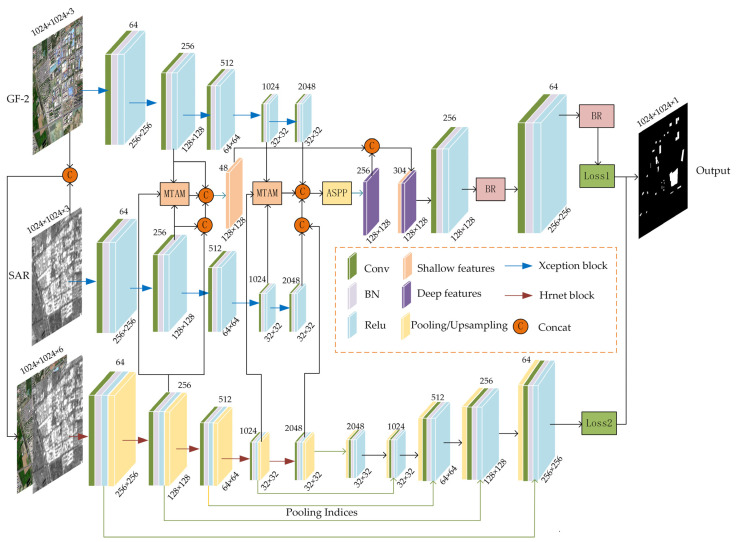
Overall structure of the multi-source data fusion network.

A specialized network architecture was developed for the precise extraction of colored steel buildings, featuring a three-branch design. The network employs dual pseudo-twin branches to independently extract features from SAR and GF-2 images, facilitating independent feature extraction and preserving detailed information within the images. The third branch processes channel-concatenated SAR and GF-2 images, emphasizing the extraction of features that focus on the semantic information of colored steel buildings fused from the two data sources. However, in comparison with the extraction performed by the two previous branches, this approach may impact the image reconstruction in the decoding section, leading to less effective restoration of fine details within the images. Therefore, in the encoding phase, the features are integrated from the three branches to ensure the preservation of detailed information while enhancing the extraction of semantic information related to colored steel buildings. The overall structure of the multi-source data fusion network proposed in this study is illustrated in Figure 3. Xception serves as the foundational network for feature extraction from individual SAR and GF-2 images [35,36]. For the combined data branch, HRNet is the selected backbone, and ‘unpooling’ is the method used for upsampling in the decoding segment. The encoder adjusts the spatial and channel dimensions of the feature maps to synchronize the branches, which aids in the seamless integration of shallow and deep features. Shallow features are refined through a multimodal three-branch attention module (MTAM), preserving the original data through channel concatenation. Deep features are generated by fusing later-stage features from each branch through the MTAM, followed by an atrous spatial pyramid pooling (ASPP) module application. The decoder upsamples these features to match the original image scale, with a boundary refinement (BR) module enhancing edge clarity. The sequential presentation that follows details the backbone network structures and the designed modules for extracting colored steel buildings.

**Figure 4:**
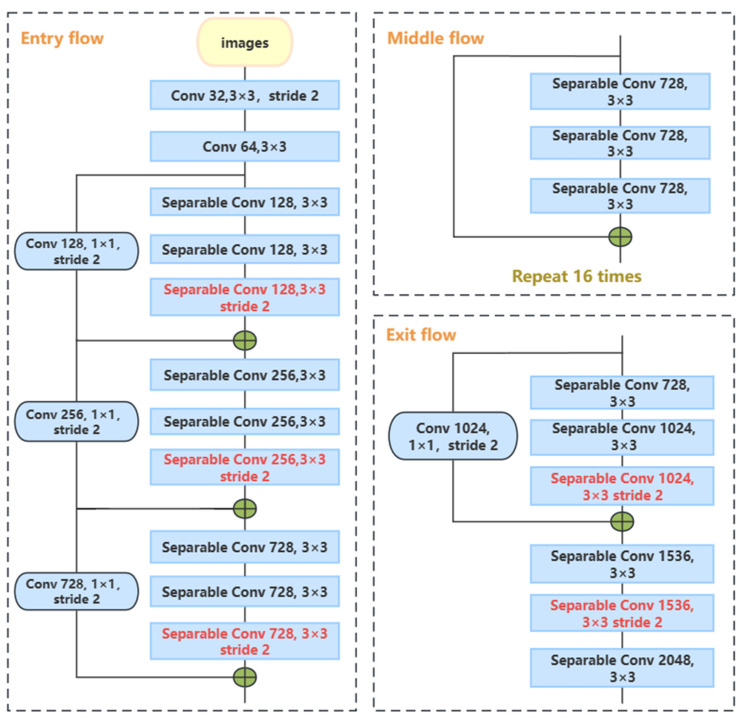
Xception network architecture.

The Xception architecture, employed for processing SAR and GF-2 images, is depicted in Figure 4 and comprises three main sections: the input, the middle flow, and the exit flow. Batch normalization (BN) is applied across all convolutional layers, including separable convolutions [37]. The core feature of Xception is its reliance on depthwise separable convolutions, which supplant traditional convolution layers. This approach iterates 16 times in the middle flow [38]. Depthwise separable convolutions streamline the convolution process by first executing spatial convolutions independently for each input channel, followed by a 1 × 1 convolution that fuses the channels [39,40]. This stratification minimizes parameters, boosting both the efficiency and the ability of the network to extract relevant features from colored steel buildings. Moreover, it diminishes data redundancy, mitigates noise, and enhances the model’s resilience to image disturbances. The model demonstrates superior adaptability and a heightened capacity for generalization in varied scenarios, such as in distinguishing colored steel building blocks.

**Figure 5:**
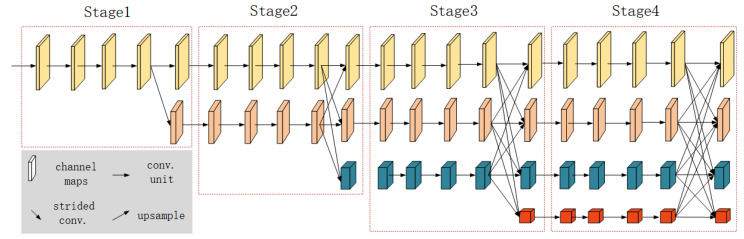
HRNet network structure.

The third branch of the network, which integrates SAR and GF-2 images, utilizes HRNet as its backbone, as shown in Figure 5. Unlike other common networks such as ResNet and VGGNet that encode images into low-resolution representations through a sequential arrangement of convolutional layers [41,42,43], HRNet adopts a parallel processing framework. This network maintains high-resolution pathways throughout, enabling the simultaneous handling of multiple resolutions. HRNet’s unique architecture processes each resolution within separate branches, continuously fusing the features across these resolutions. This method preserves global context from the lower resolution branches while capturing fine details from the higher resolution pathways. By maintaining a high-resolution representation throughout, HRNet provides a more nuanced feature map, enhancing the network’s ability to interpret semantic relationships within the image [44,45]. The adoption of HRNet thus contributes to a more detailed and accurate extraction of colored steel buildings, leveraging both global and local information.

**Figure 6:**
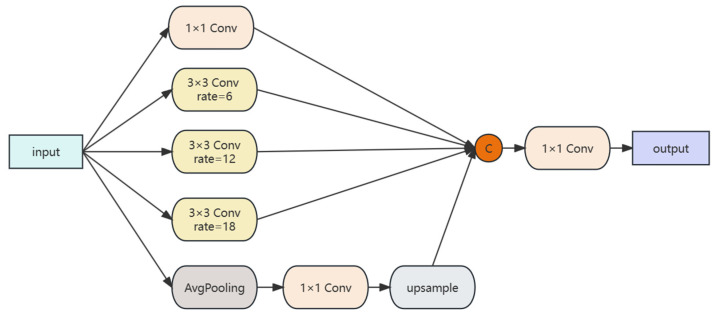
ASPP module.

The ASPP module, a concept used in DeepLabv3, is integrated to classify image regions of varying scales and to aggregate contextual information across multiple scales for robust feature extraction. Figure 6 illustrates the ASPP structure, where the feature maps undergo 1 × 1 convolution, dilated convolutions at three different rates, and adaptive average pooling. The pooled features are then upsampled to the original dimension and concatenated with the outputs from other convolutions. A final 1 × 1 convolution adjusts the channel dimensions to the desired count. This methodology enables ASPP to expand the network’s field of view and adapt to colored steel buildings of varying sizes, enhancing the network’s depth feature interpretation [46].

**Figure 7:**
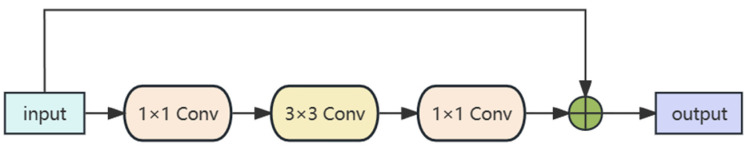
BR module.

The BR module, depicted in Figure 7, is built on residual modules. It utilizes residual connections to focus on learning the deviations between the output and input features, especially the subtle variations at the edges. This approach strengthens the delineation of edges, which is crucial for precise boundary detection in colored steel building extraction.

#### 2.4.2. Integration of the Multimodal Hybrid Attention Module

To optimally integrate features from the three branches, an MTAM was devised, combining a three-branch cross-attention module (TBCAM) and a channel attention module (CAM). The MTAM was designed to merge multi-level features efficiently, allowing the network to discern the significance of each branch’s input for specific tasks or scenarios, thereby improving the feature fusion for colored steel building extraction.

**Figure 8:**
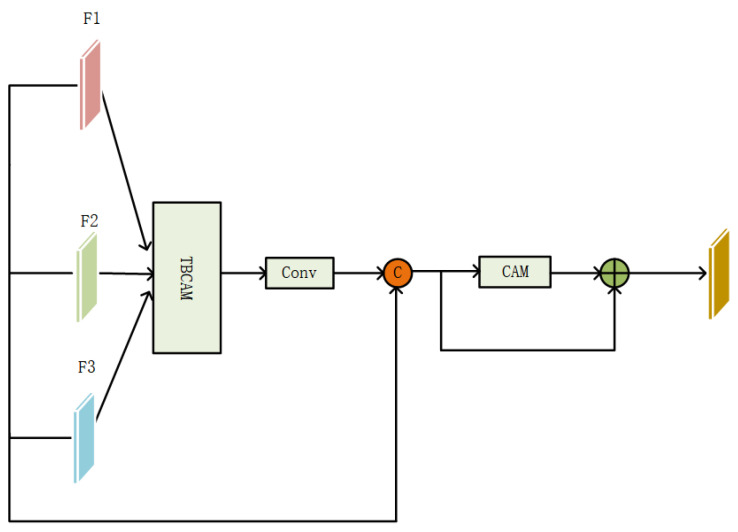
Structure of the MTAM.

As depicted in Figure 8, the GF-2 and SAR branches initially process their respective data through dedicated convolutional layers, BN layers, and activation functions. The features from these branches are then concatenated along the channel dimension, creating a composite feature that captures information from both GF-2 and SAR images. This composite feature forms the third branch, which undergoes further convolution and activation operations to reduce dimensionality and enhance the semantic information. The three branches, at a matched dimensional level, forward their features to the TBCAM. This module effectively captures spatial correlations among the branches’ features, enriching the contextual understanding. The refined features, along with the original features, are then merged and directed through the CAM. This stage harnesses the spatial and semantic context from the original data, ensuring that the semantic content on the enhanced feature channels is preserved without loss of information. The MTAM thus facilitates a comprehensive understanding of the data, which is crucial for the nuanced extraction of colored steel buildings.

**Figure 9:**
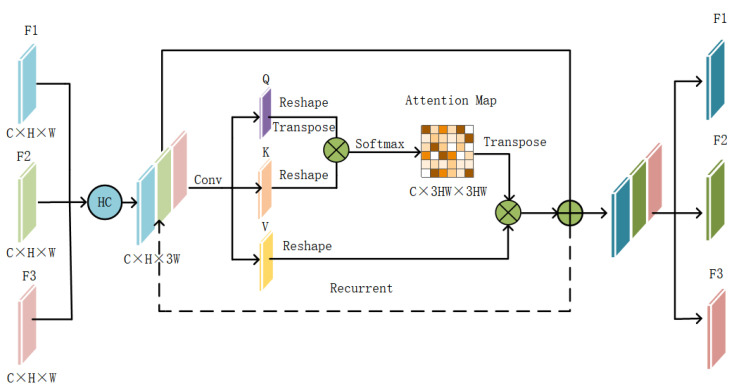
Structure of the TBCAM.

Figure 9 presents the TBCAM’s structure, utilizing feature sets $\left\{F1,F2,F3\right\}\in R^{C\times H\times W}$ from three branches as inputs. C denotes the number of channels, while H and W are the feature map’s height and width, respectively. Considering the importance of context in multi-source fusion for semantic segmentation, three new feature maps $\left\{Q,K,V\right\}\in R^{C\times H\times W\times 3}$ are derived by horizontally combining the branch features and processing them through 1×1 convolutional layers. Subsequently, each channel’s elements in the feature maps Q, K, and V are rearranged into one-dimensional vectors. Feature map Q undergoes transposition to form $Q^{T}\in R^{C\times 3HW\times 1}$ for computing attention scores. At this stage, the feature map integrates mixed features from the branches. The multiplication of $Q^{T}$ and K followed by Softmax normalization yields attention scores ranging from 0 to 1, forming the basis of the attention map. This process enhances the network’s ability to emphasize the most relevant features for accurate segmentation.

(1) $$AM_{ij}=Softmax(Q_{i}{}^{T}\times K_{j})$$

For $AM_{ij}\in R^{C\times 3HW\times 3HW}$, higher AM values indicate stronger relationships. The process continues by multiplying the vector V by the AM’s attentional weights to produce an output. This output is then added to the corresponding areas of the original spliced feature maps, resulting in enhanced feature maps. These maps are sent back through the TBCAM module to refine the features further, culminating in the final enhanced features $\left\{F1^{\prime },F2^{\prime },F3^{\prime }\right\}\in R^{C\times H\times W}$. These features effectively incorporate the contextual information from the original GF-2 and SAR data, improving the network’s segmentation accuracy through recursive refinement.

**Figure 10:**
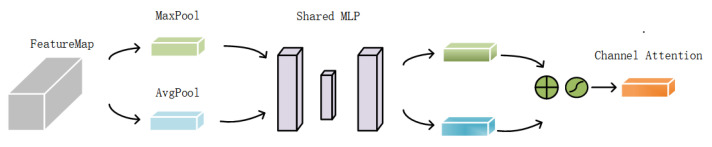
Structure of the CAM.

The CAM is depicted in Figure 10. It takes the enhanced features $\left\{F1^{\prime },F2^{\prime },F3^{\prime }\right\}\in R^{C\times H\times W}$ from the TBCAM and the original features $\left\{F1,F2,F3\right\}\in R^{C\times H\times W}$ of the three branches as inputs. The CAM’s role is to apply adaptive weights to these feature channels, directing focus to the most informative ones. To efficiently generate channel attention features, spatial dimensions of the feature map are compressed. Spatial information is commonly aggregated through average pooling, but max pooling is also utilized to highlight distinctive object features, thereby achieving more focused channel attention. After pooling, the features undergo channel splicing, and a multilayer perceptron (MLP) learns the inter-channel relationships and weights, producing two sets of channel attention vectors. These vectors are then combined and passed through a sigmoid function σ, resulting in the final channel attention feature map. This map effectively amplifies the most relevant channel features, enhancing the model’s accuracy in identifying colored steel buildings. The specific formula is as follows:

(2) $$F_{CA}=\sigma (MLP(AvgPool(F))+MLP(MaxPool(F))).$$

where F represents the input labeled FeatureMap in Figure 10 below; Avgpool and MaxPool represent average pooling and max pooling, respectively.

#### 2.4.3. Implementing Deep Supervision for Enhanced Training

Figure 3 illustrates a deep, multi-branch network structure that is susceptible to diminishing gradients during backpropagation, which can complicate training. To address this, a deep supervision strategy is employed, which incorporates auxiliary loss functions at various network layers. These auxiliary loss functions, referred to as Loss2, assist in preserving the original information and augment the model’s prediction accuracy for the input data. Working in tandem with the main loss function, Loss1, the auxiliary loss functions bolster the training process and enhance the network’s generalization capabilities on new, unseen data. Additionally, an inverse pooling technique is used in the decoding stage of the third branch, utilizing saved indices from the prior maximum pooling steps. This method helps restore the location and detail information lost during pooling, leading to more accurate pixel-level output and refined capture of intricate input data features.

## 3. Results

### 3.1. Experimental Setup

Experiments were conducted using the PyTorch framework (version 1.8.1) for building neural network models, supported by four NVIDIA RTX3090 GPUs, each with 24 GB of memory, to enhance training efficiency. Two loss functions, cross entropy (CE) and dice coefficient (DICE), were chosen due to CE’s compatibility with optimization methods like gradient descent and DICE’s effectiveness in handling class imbalances. The Adam optimizer was employed with an initial learning rate set to 0.00003 and a warm-up phase for the initial 500 iterations. Weight decay was set at 0.001 to prevent overfitting.

### 3.2. Evaluation Metrics

Assessment of the model’s performance relies on metrics calculated from the confusion matrix. Initially, predictions for colored steel buildings within the Beijing–Tianjin–Hebei region were made. These predictions were then manually verified and corrected to serve as accurate labels for evaluation. We employed two widely recognized evaluation metrics: intersection over union (IoU) and F1 score.

IoU is a measure that calculates the ratio of overlap to the combined area of predicted and actual values for a specific category. It reflects how much the predicted area coincides with the actual area and can be expressed as follows:

(3) $$IOU=\frac{TP}{TP+FN+FP}$$

Precision measures the proportion of accurately classified pixels within the prediction results, while recall signifies the percentage of correctly predicted pixels among those identified as colored steel building results. The F1 score is defined as the harmonic mean of precision and recall, effectively harmonizing the two into a single metric. Equations (4) and (5) provide the calculations for recall and precision, respectively, while Equation (6) presents the formula for computing the F1 score, as shown below:

(4) $$Recall=\frac{TP}{TP+FN}$$

(5) $$Precision=\frac{TP}{TP+FP}$$

(6) $$F1=2\times \frac{Precision\times Recall}{Precision+Recall}$$

In the aforementioned formulas, TP denotes pixels correctly identified as colored steel buildings, FP represents pixels labeled as background but falsely detected as colored steel buildings, and FN signifies pixels labeled as colored steel buildings but erroneously detected as background.

### 3.3. Ablation Study

An ablation study was performed using data from the GF-2 and Sentinel-1 satellites to assess the effectiveness of the proposed modules in recognizing colored steel buildings. We selected the deep learning model DeepLabv3+ as the baseline network and Xception as the backbone network. Subsequently, we examined the impact of incorporating SAR data through the proposed network branches and modules on the model’s accuracy. Table 1 details the outcomes in terms of four evaluation metrics obtained after sequentially integrating the network components. Figure 9 illustrates the colored steel building extraction results from the test dataset, showcasing the model’s practical application.

Table 1 summarizes the performance enhancements achieved by incorporating additional branches and modules into the proposed network. The baseline DeepLabv3+ network registered an F1 score of 76.68% with the selected dataset. By fusing the SAR and GF-2 data’s RGB bands and training with DeepLabv3+, the F1 score improved marginally to 77.23% for the test group. Altering the network to a dual-branch structure with GF-2 and SAR data raised the F1 score to 78.25%, demonstrating that the method of data fusion significantly affects the outcome. Further adding a third branch of channel-spliced GF-2 and SAR data, along with corresponding feature splicing across the three branches, elevated the F1 score to 79.98%. Incorporating the depth supervision strategy and MTAM into the three-branch network structure resulted in F1 scores of 80.79% and 82.60%, respectively, both demonstrating accuracy improvements. However, the MTAM module proved to be more effective. Therefore, by simultaneously incorporating the MTAM module and depth supervision strategy into the three-branch network, our final configuration achieved an F1 score of 83.19%. While the improvement over solely adding the MTAM module was only a slight enhancement, it marked an approximate six-point gain over the baseline model.

**Figure 11:**
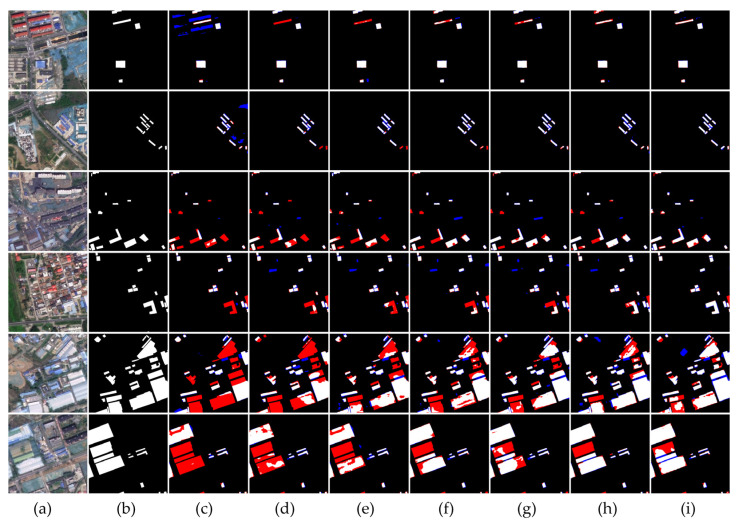
Examples of different scenarios of ablation experiment visualization result images. (**a**) Original image; (**b**) Labeled values; (**c**) DeepLabv3+; (**d**) DeepLabv3+ (RGBS); (**e**) DeepLabv3+ (Dual Branch); (**f**) DeepLabv3+ (Three Branches); (**g**) DeepLabv3+ (Three Branches depth supervision); (**h**) DeepLabv3+ (Three Branches)-MTAM; (**i**) Ours.

Figure 11 displays a comparative analysis of colored steel building extraction across various scenarios. The first column presents images from the test group, the second column shows the corresponding labels, and the third column illustrates the extraction results from the baseline DeepLabv3+ network. The subsequent columns demonstrate the progressive improvement in results when the proposed branches and modules for SAR data fusion were added to the baseline network. The results are visually annotated to indicate errors: red signifies missed detections of colored steel buildings and blue indicates areas incorrectly identified as such. The ablation study’s outcomes in Figure 11 are organized into three scenario-based groups. The first two rows depict sparse colored steel building scenarios; rows three and four show instances of small, more dispersed colored steel buildings; and the last two rows illustrate densely distributed large colored steel buildings. In scenarios with sparse colored steel buildings, the baseline DeepLabv3+ network tends to incorrectly identify features of a similar shape and color as colored steel buildings. The gradual incorporation of this network’s modifications demonstrates a reduction in these errors, pointing to the effectiveness of the proposed data fusion and network architecture enhancements.

In the first set of results, the baseline network DeepLabv3+ often mistakes residential buildings for colored steel buildings due to their similar appearance. However, the six subsequent SAR image fusion techniques introduced in this study reduce these errors. The false detection rates decrease progressively from the channel fusion approach shown in Figure 11c to the two-branch fusion in Figure 11d, and further to the three-branch fusion in Figure 11e. By the stage shown in Figure 11i, the model achieves near elimination of leakage, and edge definition shows noticeable enhancement compared to Figure 11h. In the second row, representing construction sites with colors akin to colored steel buildings, the likelihood of false detections is high. While the channel fusion approach using SAR data better captures the characteristics of colored steel buildings and lessens false positives, it also results in some leakage. Nonetheless, as the fusion method is refined and additional modules are integrated, both the leakage and false detection rates see considerable improvements.

In the second set of results, which features small, dispersed colored steel buildings in complex scenes with various similar structures, the baseline DeepLabv3+ network experiences significant leakage, indicating missed detections. However, adopting the three-branch fusion approach reduces this leakage. Introduction of the MTAM and the deep supervision strategy further mitigates leakage by effectively focusing on the pertinent features of colored steel buildings. In the fourth row, where the scene is cluttered with buildings resembling colored steel structures, the baseline DeepLabv3+ network struggles to accurately discern the colored steel buildings, leading to leakage and a few false detections. The integration of SAR data helps to reduce leakage but introduces many false positives due to the color similarity between colored steel buildings and nearby structures. The combined application of the MTAM and the deep supervision strategy enhances the model’s attention to contextual scene information, improving its ability to recognize the distinctive distribution characteristics of colored steel buildings and thereby reducing both false detections and leakage. Despite these advancements, the complexity of scenes containing numerous buildings presents challenges, including potential labeling inaccuracies during sample creation. Consequently, several extraction results may contain minor errors. This area, particularly the judgment difficulty in dense building environments, warrants further research and methodological refinement.

In the third group of colored steel building extraction results, there is some irregular distribution of large-area colored steel buildings; the baseline network cannot easily learn the characteristics of the colored steel buildings, resulting in the extraction of the results being incomplete. In the third group of results, DeepLabv3+ has serious leakage. The network of channel-splicing fusion SAR reduces the leakage, but the effect is not significant enough; with the introduction of network branching fusion, the SAR network effect has gradually improved. After the introduction of the MTAM module and deep supervision strategy, the neural network can be adjusted to pay attention to different data sources by calculating the weights of different parts or features of the input, which reduces the leakage and misdetection to a certain extent, so as to improve the results of the extraction of colored steel buildings.

### 3.4. Method Comparison

To ascertain the superiority of the proposed approach for semantic segmentation, comparative experiments were conducted, with conventional methods applied to GF-2 images and combined GF-2 and SAR images. The comparative set includes DeepLabv3+, UNet, UNet++, PAN, SegNet, and Swinunet. The selected baseline, DeepLabv3+, employs the ASPP technique, which uses atrous convolution at varying rates to expand the receptive field and grasp multiscale contextual information. UNet, a pioneer among semantic segmentation models, features an encoder–decoder structure with skip connections to preserve details. UNet++ builds on this by adding denser skip connections to further refine segmentation precision. PAN leverages a pyramid network structure with an attention mechanism to integrate semantic information across scales, enhancing detail through attention-guided recovery. SegNet improves edge definition by using the indices from the max-pooling layers for non-linear upsampling, thus reducing the model’s parameter count. Swinunet introduces a Swin-Transformer-based architecture, segmenting input feature maps into small windows and applying self-attention within these windows to learn localized features effectively.

**Figure 12:**
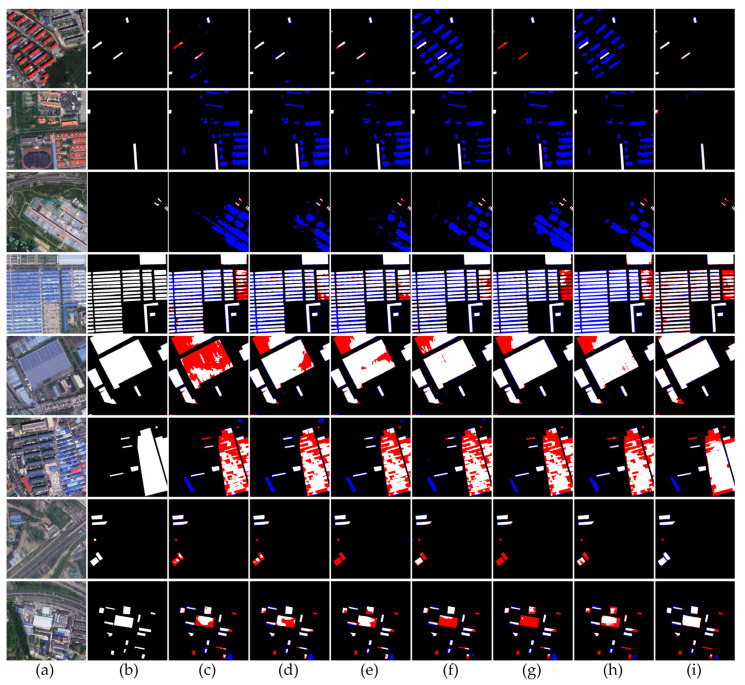
Comparison images for six GF-2 and SAR channel-fused semantic segmentation networks. (**a**) Original graph; (**b**) Labeled values; (**c**) DeepLabv3+ (RGBS); (**d**) Unet (RGBS); (**e**) UNet++ (RGBS); (**f**) PAN (RGBS); (**g**) SegNet (RGBS); (**h**) Swinunet (RGBS); (**i**) Ours.

Table 2 shows that the proposed method outperforms other semantic segmentation approaches. The baseline network DeepLabv3+ has an F1 score of 76.68%, with other methods scoring similarly around 77%. The introduction of SAR data via channel fusion results in a slight improvement to precision. However, the extent of the precision enhancement varies with different fusion techniques. The multi-source data fusion network suggested in this study demonstrates a significant boost in both precision and recall, with the F1 score reaching 83.19%. In Figure 12, the results indicate superior extraction performance across various scenarios when compared to other networks, especially in complex scenes with materials that could be confused with colored steel buildings. The comparison demonstrates that the method achieves more accurate results, with fewer instances of false detection.

Figure 12 presents a visual comparison of the segmentation results. The first column contains images from the test dataset, the second column displays the true labels, and the remaining columns show predictions from various networks, including those that merge GF-2 and SAR data and the specifically designed multi-source data fusion network. The comparison is divided into four scenario-based categories. The first and second rows constitute the first category, depicting environments where colored steel buildings are situated amidst similar structures. The third row forms the second category, illustrating areas where colored steel buildings are surrounded by features of a similar color or shape. Rows four to six represent the third category, showcasing larger colored steel buildings or those that are closely spaced. Finally, rows seven and eight depict the fourth category, where small- to medium-sized colored steel buildings are sparsely distributed. This categorization helps to highlight the efficiency of the proposed fusion network in differentiating colored steel buildings from similar structures across a range of complex scenarios.

In the first category of results, where colored steel buildings are mixed with similar structures, conventional semantic segmentation networks tend to make numerous misdetections with few omissions. However, the network with the MTAM shows a marked reduction in both misdetections and omissions. In the first scenario, residential buildings with colors and shapes akin to colored steel buildings often lead to confusion. Networks like PAN and Swinunet incorrectly identify all residential buildings as colored steel structures. Although SegNet experiences leakage without misdetection, other mainstream networks also suffer from minor misdetections and leakage. The network incorporating the MTAM better learns the features specific to colored steel buildings from both datasets, resulting in more accurate extractions. In the second scenario, roofs of tile and concrete, which are similar in color and shape to colored steel buildings, are often misclassified by mainstream networks due to the overlapping of optical and SAR data at the image level, which causes feature extraction interference. In contrast, the designed multi-source data fusion network captures more distinctive and representative features from optical and SAR data. This effectively leverages the information from buildings made of various materials, reducing false detections in similar scenes.

In the second set of scenes, where small colored steel buildings are present, mainstream networks continue to exhibit a high rate of misdetection. Our network, however, significantly minimizes such errors, albeit with many instances of leakage. Specifically, in the third row, where a colored steel building is situated near a construction site, the large concrete areas resemble the blue-colored steel buildings, leading to misdetection by all other networks. The multi-source data fusion network designed for this study does not falsely identify construction sites as colored steel buildings, even though it may miss a few small colored steel structures. This distinction suggests that the network is capable of effectively discerning material differences in complex urban environments.

In the third group of scenes, colored steel buildings with varying gap sizes are labeled differently during the sample annotation process. For the fourth row, colored steel buildings with large gaps but dense distribution are labeled as single units, while those with minimal spacing in the fifth and sixth rows are grouped together. Other segmentation methods exhibit significant omissions when identifying large expanses of colored steel buildings. Specifically, in the fourth row, they tend to falsely identify gaps between buildings as part of the structures, whereas the BR module in the proposed network enhances edge optimization, allowing for distinct extraction of colored steel buildings with larger gaps. In the fifth row, other networks miss large areas of colored steel buildings, with DeepLabv3+ showing significant omissions and comparative networks displaying minor ones. Our network, however, with its advanced learning capabilities, reduces these omissions by better recognizing the features of colored steel buildings. For the diverse colored steel buildings in the sixth row, our network diminishes leakage in this complex scene. Current networks struggle to extract colored steel buildings based solely on color and high-resolution imagery. Although SAR image fusion assists in this task, it does not entirely prevent a small number of false detections. Nonetheless, our network achieves a relative decrease in such errors.

In the fourth group of scenarios, characterized by sparsely distributed small- and medium-sized colored steel buildings, common networks often fail to completely extract the buildings and frequently misidentify them. In the seventh row, where only a few colored steel buildings are present, the UNet++ and SegNet networks overlook them, while other networks result in partial extractions. In the eighth row, which contains colored steel buildings of varying sizes, the proposed network does not fully capture the smaller buildings, resulting in some leakage and misdetection. However, for medium-sized colored steel buildings in this row, the PAN and SegNet networks perform poorly in their extraction, and while other networks do manage to extract them, the detailing of the edges is inadequate. In contrast, our network efficiently extracts medium-sized colored steel buildings without any missed detections and with significantly improved edge detailing compared to other networks.

### 3.5. Comparison of Attention Modules

In this research, various attention modules were evaluated. The MTAM was specifically designed to discern the significance of each branch in diverse scenarios. This facilitates the integration of optical and SAR data, thus enhancing the accuracy of colored steel building extraction. Traditional attention modules typically handle single inputs, and many manage dual inputs through hybrid attention. We compared the MTAM with the conventional single-branch dual attention network (DANet) attention module and multimodal cross-attention module (MCAM). DANet employs self-attention to identify spatial and channel feature dependencies, using position and channel attention to improve feature discrimination in scene segmentation. The MCAM focuses on the spatial relationships within single-data-source feature maps and interacts with SAR and optical image features to understand the relationships between dual input features. The MTAM merges the strengths of both these approaches within a three-branch multi-source data fusion network structure. It processes three branches of features through the attention module, capturing correlations in both the channel and spatial dimensions, thus more effectively highlighting the importance of features across each branch. We replaced the MTAM of the proposed multi-source data fusion network by integrating DANet to each branch as a comparison experiment. Similarly, we put the SAR and GF-2 dual-branch feature fusion through the MCAM attention module. The findings, as detailed in Table 3, indicate that the precision of the fusion method using the MTAM surpasses that of the other two attention modules.

### 3.6. Cartography of Colored Steel Buildings in the Beijing–Tianjin–Hebei Region

**Figure 13:**
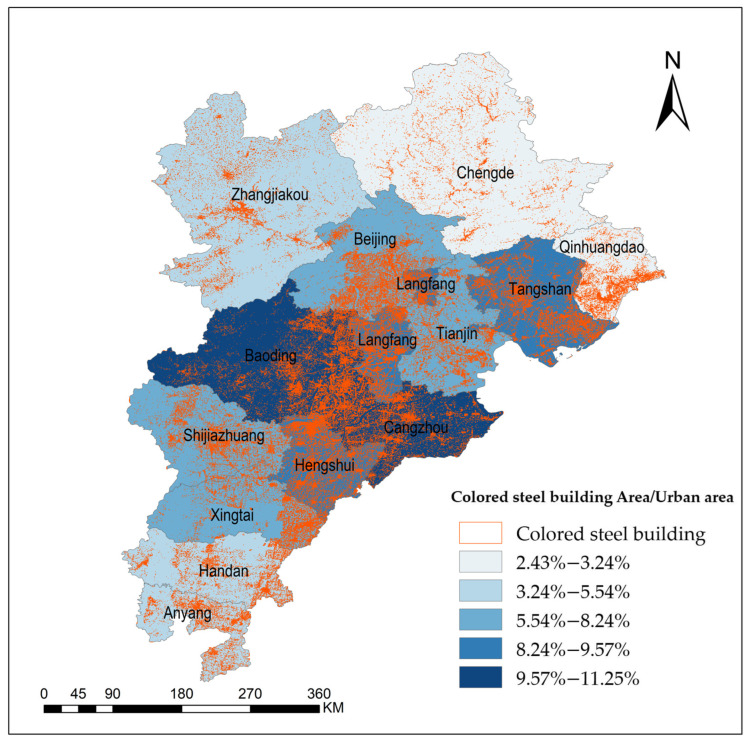
Mapping results for colored steel buildings in the Beijing–Tianjin–Hebei region of China based on 0.8 m satellite imagery. Orange areas represent the vector patches of colored steel buildings. Blue areas with different depths represent the proportion of the city’s colored steel building area in the city cluster.

The proposed network structure was employed to map colored steel buildings at a 0.8 m resolution within the Beijing–Tianjin–Hebei urban cluster. The results are illustrated in Figure 13, where orange vector patches represent the extracted colored steel buildings. A blue gradient scale indicates the proportion of each city’s colored steel building area relative to the total within the entire cluster. This proportion is categorized into five levels of increasing intensity from light to dark. The data reveal that colored steel buildings are predominantly located in Baoding and Cangzhou, with each city’s area making up between 9.57% and 11.25% of the cluster’s total. Langfang, Tangshan, and Hengshui follow, each with a share of 8.24% to 9.57%. Centralized distributions are seen in Beijing, Tianjin, Shijiazhuang, and Xingtai, with their shares ranging from 5.54% to 8.24%. Zhangjiakou, Xingtai, and Handan have a sparser presence, with shares between 3.24% and 5.54%. Chengde and Qinhuangdao have the smallest shares, with only 2.43% to 3.24% each.

## 4. Discussion

### 4.1. Comparison of Fusion Methods

Common fusion methods include decision-level fusion, data-level fusion, and feature-level fusion. The choice of these fusion methods depends on the task requirements, data properties, and application scenarios. Therefore, in this section, we compare these fusion methods and select the one suitable for the steel building task, with precision values shown in Table 4. Using only high-resolution images as inputs to the DeepLabv3+ baseline network, we achieved an F1 accuracy of 76.68%. Decision-level fusion combines different decisions from different networks. Therefore, we evaluated the results obtained separately by inputting GF-2 and SAR into the baseline network, with a weighted sum. Precision showed a slight decrease and recall showed a slight improvement, but the F1 accuracy did not improve significantly. Data-level fusion first merges data and then uses the merged data for the task. We stacked the GF-2 and SAR channel dimensions as inputs to the network, resulting in a slight improvement in F1 accuracy. In addition, we achieved an F1 accuracy of 77.46% by combining the two decision results obtained separately from training GF-2 and SAR with the GF-2 and SAR images in the channel dimensions. Overall, the decision-level fusion method did not show a significant improvement on our dataset. Feature-level fusion connects feature vectors from different branches. By inputting GF-2 and SAR through two different branches as the network’s input, we achieved an F1 accuracy of 78.25%, showing a noticeable improvement in accuracy. Therefore, we further explored the feature-level fusion method, designing a three-branch multi-source data fusion network and ultimately achieving good results.

### 4.2. Selection of a More Suitable Backbone

In this research, we chose HRNet as the backbone network for the third branch formed by the concatenation of SAR images and GF-2 images in the channel. HRNet itself can retain high-resolution feature maps simultaneously at different stages, allowing the network to capture multiscale information. To better validate the effectiveness of introducing the third branch in our network, which aids in handling detailed image information, we replaced the third branch with other common backbones for comparative experiments. The backbones used for comparison include ResNet, EfficientNet, and Swin Transformer. As shown in Table 5, HRNet is more suitable for our proposed multi-source data fusion network, demonstrating superior performance in capturing detailed information regarding colored steel structures and handling multiscale features. It is better suited for the scenario of colored steel building extraction.

## 5. Conclusions

The effective identification of colored steel buildings in images plays a vital role in managing the construction industry and promoting urban sustainability. Traditional remote sensing methods, or those relying solely on optical imagery via deep learning, often struggle to differentiate building materials accurately. In this study, we introduced SAR data and presented a multi-source data fusion network tailored for colored steel building extraction. The network architecture consists of three branches: two are pseudo-twin structures processing GF-2 and SAR data independently, and the third combines features from GF-2 and SAR channel fusion. This tri-branch setup independently extracts features from varied data sources, preventing information loss or interference that could occur from merging at the image level. It also allows parameter adjustments within each branch to cater to diverse and complex scenarios, aiming to reduce issues like overfitting or underfitting. A novel MTAM was designed to direct the network’s focus to different input segments, thus enhancing the contextual understanding of the data. Additionally, a deep supervision strategy with an auxiliary loss function was implemented in the third branch to facilitate more effective learning of feature representations.

Ablation and comparative experiments were performed to assess the proposed network branches and modules. These experiments demonstrate that various fusion methods of the GF-2 and SAR data lead to different levels of accuracy. The designed multi-source data fusion network was validated with an F1 score of 83.19%, achieved on the curated colored steel building dataset. Comparative tests with established semantic segmentation networks also support the enhancement of extraction results through the integration of SAR data. However, there are imperfections in the current network design. Firstly, the resolution disparity between Sentinel-1 SAR images and high-resolution images necessitates resampling, which may result in information loss. Secondly, while SAR imagery serves as auxiliary data by which to distinguish the materials of colored steel buildings, there is a spectrum of multi-source data fusion methods available, and alternative network configurations might yield superior outcomes. Future research will explore the use of higher resolution SAR data in conjunction with optical data and will investigate different multi-source data fusion network structures to potentially enhance the accuracy of colored steel building extractions.

## Figures and Tables

**Table 1:** Results of ablation experiments.

Method	Metrics (%)			
	Precision	Recall	F1	IoU
DeepLabv3+	75.26	78.15	76.68	62.17
DeepLabv3+ (RGBS)	76.07	78.44	77.23	62.91
DeepLabv3+ (Dual Branch)	77.47	79.04	78.25	64.27
DeepLabv3+ (Three Branches)	80.97	79.02	79.98	66.64
DeepLabv3+ (Three Branches)-Deep Supervision	82.30	79.34	80.79	67.77
DeepLabv3+ (Three Branches)-MTAM	84.27	81.01	82.60	70.36
Ours	84.16	82.24	83.19	71.21

**Table 2:** Comparison of experiment results.

Method	Metrics (%)			
	Precision	Recall	F1	IoU
DeepLabv3+	75.26	78.15	76.68	62.17
UNet	77.13	77.34	77.24	62.91
UNet++	77.34	77.19	77.27	62.95
PAN	76.70	78.08	77.38	63.11
SegNet	76.83	78.03	77.42	63.16
Swinunet	77.14	77.11	77.13	62.77
DeepLabv3+ (RGBS)	76.07	78.44	77.23	62.91
UNet (RGBS)	77.97	78.02	77.99	63.86
UNet++ (RGBS)	77.87	77.79	77.83	63.65
PAN (RGBS)	80.61	79.07	79.84	66.44
SegNet (RGBS)	80.42	76.34	78.33	64.37
Swinunet (RGBS)	79.79	75.07	77.36	63.08
Ours	84.16	82.24	83.19	71.21

**Table 3:** Comparison of different attention modules.

Method	Metrics (%)			
	Precision	Recall	F1	IoU
DANet [47]	80.91	80.21	80.55	67.44
MCAM [27]	81.58	80.70	81.14	68.26
Ours	84.16	82.24	83.19	71.21

**Table 4:** Comparison of different fusion methods.

Method	Metrics (%)			
	Precision	Recall	F1	IoU
Baseline	75.26	78.15	76.68	62.17
Decision-level fusion	72.29	81.70	76.71	62.22
Data-level fusion	76.07	78.44	77.23	62.91
Decision-data fusion	75.06	80.02	77.46	63.21
Feature-level fusion	77.47	79.04	78.25	64.27

**Table 5:** Comparison of different backbones.

Method	Metrics (%)			
	Precision	Recall	F1	IoU
ResNet	82.77	80.19	81.46	68.72
EfficientNet	82.40	80.18	81.28	68.46
Swin Transformer	82.46	79.47	80.94	67.98
Ours	84.16	82.24	83.19	71.21

## Data Availability

The data are not publicly available due to currently proprietary.
